# Well-Being, Obesity and Motricity Observatory in Childhood and Youth (WOMO): A Study Protocol

**DOI:** 10.3390/ijerph17062129

**Published:** 2020-03-23

**Authors:** María Mendoza-Muñoz, José Carmelo Adsuar, Jorge Pérez-Gómez, Laura Muñoz-Bermejo, Miguel Ángel Garcia-Gordillo, Jorge Carlos-Vivas

**Affiliations:** 1 Health, Economy, Motricity and Education Research Group (HEME), Faculty of Sport Sciences, University of Extremadura, 10003 Cáceres, Spain; mamendozam@unex.es (M.M.-M.); jorge.carlosvivas@gmail.com (J.C.-V.); 2 Social Impact and Innovation in Health (InHEALTH), University of Extremadura, 10003 Cáceres, Spain; lauramunoz@unex.es; 3 Facultad de Administración y Negocios, Universidad Autónoma de Chile, sede Talca 3467987, Chile; miguelgarciagordillo@gmail.com

**Keywords:** body max index, fitness, lifestyle, literacy, motor-skills, obese, physical condition, self-perceived, strength

## Abstract

Background: Childhood obesity is one of the greatest public health problems facing advanced societies, and Spain is one of the countries with the highest incidence. There are many studies that monitor at the national level, but given the lack of specificity, lack of updating and scarcity of epidemiological data on overweight, obesity, physical condition and well-being of children and adolescents in Extremadura, it seems necessary to create a Well-being, Obesity and Motricity Observatory in Childhood and Youth (WOMO) in Extremadura in order to follow up on the evolution of this problem and to propose strategies to combat it. Therefore, this project aims (1) to obtain information on the physical condition, body composition and well-being of children and youth in Extremadura every year and (2) to evaluate the relationship between obesity, overweight, physical condition, well-being, self-perceived physical fitness, physical activity adherence, physical literacy, health-related quality of life (HRQoL), happiness and satisfaction with life, connection with nature, barriers to sports practice, self-concept and bullying in children and youth. Methods: An annual monitoring cross-sectional and follow-up study will be performed. Primary outcome measures will be (1) personal data and anthropometric measurements, (2) family and school information, (3) physical condition, (4) obesity and overweight level, and (5) well-being. Secondary outcome measures will be (1) self-perceived physical fitness, (2) physical activity adherence, (3) physical literacy, (4) HRQoL, (5) happiness and satisfaction with life, (6) connection with nature, (7) barriers to sports practice, (8) self-concept and (9) bullying. Discussion: This project will provide valuable information to adopt appropriate strategies to reduce the level of overweight and obesity in children and youth. Furthermore, orientations will be given to transfer the results obtained to the business sector or to the public sector to evaluate or change the policies adopted.

## 1. Introduction

Childhood obesity is one of the greatest public health problems facing advanced societies [1]. Spain is one of the countries with the highest incidence of overweight and obesity in the world [2]. Several studies have aimed to determine the obesity and overweight levels in child and youth populations. In order to monitor the overweight and obesity values as well as the lifestyles of Spanish child population, the Study of Growth Monitoring, Nutrition, Physical Activity, Child Development and Obesity in Spain (ALADINO) was born [3,4], which provides data to the European initiative Childhood Obesity Surveillance Initiative (COSI) (led by the Spanish Agency for Food Safety and Nutrition, AESAN, of the Ministry of Health, Consumption and Social Welfare). Specifically, this study informed that 41.3% of children aged between 6 and 9 years (42.8% boys vs. 39.7% girls) present overweight or obesity. However, as just mentioned, this study only includes students from 6 to 9 years old, thus leaving children and adolescents from 10 to 17 years old without reference data.

Several studies have attempted to assess the level of overweight and obesity [2,3,5,6]. In 2017, the Spanish National Health Survey (SNHS), applied to children and adolescents aged between 2 and 17 years, determined that the percentage of overweight and obesity in Spain, based on body mass index (BMI), was 28.6% considering both sexes. Moreover, there was a similar prevalence of overweight and obesity in boys and girls (18.30% vs 18.20% and 10.40% vs 10.2%, respectively). Specifically, the percentages of overweight and obesity in Extremadura were 11.88% and 10.38%, respectively. In line with national outcomes, there was also a higher prevalence of overweight and obesity in boys than girls (15.30% vs 12.69% and 8.08% vs 7.83%, respectively). However, this study does not specify the percentage or sample size proceeding from the region of Extremadura. Likewise, the last national-level study in this topic, named the PASOS study, showed 20.7% overweight and 14.2% obesity in children and adolescents aged between 8 and 16 years [5]. However, this study neither indicated the sample size of every region nor differentiated between sex. Moreover, all participating centres in the region of Extremadura were placed in Badajoz province. Thus, there are no representative data from the Cáceres province despite this area practically including the other half of the total population of Extremadura.

Regarding well-being, some studies have determined that there is a relationship between negative self-perceptions and the reduction in satisfaction with life or general well-being (affecting women more than men in relation to weight loss [7,8]), establishing a relationship and being especially important in children who are overweight or obesity [9]. In this regard, the PASOS study refers to emotional well-being, linking it to indicators of health-related quality of life (HRQoL) and determining that more than 20% of children and adolescents feel worried, sad or unhappy, affecting females more than males (25% vs. 16.6%) and more during the adolescence than childhood (25% vs. 15.1%). This could indicate that, as child and youth obesity increases, the satisfaction with life or general well-being of children proportionally decreases [5].

Another of the main problems we can find related to child and youth obesity is the high degree of sedentarism, which is considered the disease of the 21st century [10,11]. Sedentary lifestyles have been aggravated in recent years by the increased use of new technologies and are directly related to childhood obesity [12]. Thus, some recent studies have evaluated physical inactivity, reporting similar and worrying results. The PASOS study showed that only 36.7% of children and youth fulfil with the World Health Organization (WHO) recommendations for children aged between 5 and 17 years, which recommend at least 60 min of moderate or vigorous physical activity per day. Also, this study has indicated that the percentage of inactivity is higher in girls than boys (70.1% vs. 56.1%) and higher in adolescents than children (69.9% vs. 56.1%). In the same line, the 2015 ALADINO report [13] shows that around 68% of children aged 6–9 years take less than 4 h of physical activity per week. Both studies present data on a national level, without differentiating between regions.

At world level, we found the Global Matrix project, which is an international initiative where multidisciplinary teams of experts from numerous countries concurrently develop national report cards in response to increasing noncommunicable diseases related to physical inactivity among individuals of all ages [6,14,15]. This initiative is led by the Active Healthy Kids Global Alliance (AHKGA), an incorporated not-for-profit organization dedicated to improving the physical activity of children and youth worldwide. In 2018, the AHKGA released the Global Matrix 3.0, compiling report card grades from 49 different countries for 10 physical activity indicators: five behavioural indicators (overall physical activity, organized sport and physical activity, active play, active transportation and sedentary behaviour), one individual characteristic indicator (physical fitness) and four sources of influence indicators (family and peers, school, community and environment, and government) [6]. The main findings of every participating country were summarized in forty-nine short report card manuscripts. Report card results were published according to the predetermined Human Development Index (HDI) of every country (<0.70 low and medium; ≥0.70 to <0.80 high; ≥0.80 very high HDI). Global Matrix describes a score scale from A+ to F, where A+ is the best value for that indicator with 15 points. In Spain, the worst values for the indicators were physical activity (D) 5/15; active play (C−) 7/15; or school (C+) 9/15. Also, the average was (C+) 9/15 [6].

Specifically, the Spanish research group concluded that 52% males and 39.8% females from 6 to 9 years old achieved at least 60-min moderate-to-vigorous physical activity (MVPA) per day [16,17]; 31% males and 14.9% females from 3 to 18 years old completed ≥5 days per week of 60-min MVPA [18]; and 34.2% males and 26.9% females from 3 to 14 years old realized at least 60-min MVPA per day [19]. Likewise, they reported that 73.3% males and 65.6% females between 6 and 9 years old participated in organized sport and/or physical activity programs out of school [3]. Their data for most of the indicators were extracted from the ALADINO study; thus, there exists an age limitation, as we mentioned above.

Given the lack of specificity, the lack of updating and the scarcity of epidemiological data on overweight, obesity, motricity and well-being of children and adolescents in Extremadura, especially from 10 to 17 years old, it is necessary to create a Well-being, Obesity and Motricity Observatory in Childhood and Youth (WOMO) in Extremadura in order to follow up on the evolution of this problem and to propose strategies to combat it.

Likewise, into the different models of the “Health Promotion and Disease Prevention Toolkit” is crucial to include some indicators which allow to establish the background and current state about an issue as well as to observe these health indicator’s evolution over time and to assess the adopted policies’ impact to attend this issue. For instance, this proposal could fit in the standards of the Predisposing, Reinforcing and Enabling Constructs in Educational Diagnosis and Evaluation model (PRECEDE) or some components that determine the ability to identify key decision-making points that influence health behaviors, inter alia [20,21]. Specifically, this proposal would be mainly related to those factors related to epidemiological assessment of the PRECEDE model, which aims to identify the health determinants of the identified problems and to set priorities and goals [20]. It would be also associated to the component of gathering information by conducting a health needs assessments and other efforts to determine who is at risk and the population that should be targeted, included into the Health Belief Model [21].

Therefore, the main objective of this project is to obtain information on the physical condition, body composition and well-being of children and youth in Extremadura every year. Likewise, this project tries to evaluate the relationship between obesity, overweight, physical condition, well-being, self-perceived physical fitness, physical activity adherence, physical literacy, HRQoL, happiness and satisfaction with life, connection with nature, barriers to sports practice, self-concept and bullying in children and youth.

## 2. Materials and Methods

### 2.1. Study Design

An annual monitoring cross-sectional and follow-up study will be performed in order to obtain information and to monitor the health status of children and youth in the Extremadura region. In addition, the collected information will be treated with the aim of analyzing the possible relationships between the different assessed variables that could help to propose strategies for preventing overweight and obesity in children and youth.

### 2.2. Ethics Approval

Ethical approval was provided by the Bioethics and Biosafety Committee at the University of Extremadura (approval number: 138/2019; 155/2019; 139/2019).

### 2.3. Participants

A total of six primary and six secondary schools from Extremadura will be assessed for this study that supposes an estimated sample of 1000 children and adolescents (500 males and 500 females). Purposive sampling will be applied to ensure that there will be a similar number of participants regarding all age ranges.

To be included in this project, participants will meet the following inclusion criteria: (1) aged between 6 and 17 years old; (2) registered and/or lived in the autonomous community of Extremadura; (3) not suffering pathologies that contraindicate the exercise program and physical activity or that require special attention; (4) authorized by the parents or legal guardians; and (5) child or youth’s acceptance to participate in the study.

### 2.4. Measures and Procedures

A variety of tools will be used to assess physical condition, body composition and well-being of children and youth in Extremadura every year. Prior to the first measurement, all participants will carry out a familiarization phase in order to know the different instruments and evaluations included in this project (Table 1).

#### 2.4.1. Main Measures

The following questionnaires have been adapted and designed based on the models proposed by the WHO for the development of the COSI initiative [2] and ALADINO report [1]:

1. **Examiner’s questionnaire:** This questionnaire collects personal data information and anthropometric measurements from children and adolescents.
  - *Personal data.* Similarly to the ALADINO study [3], date of birth, sex, place of residence, grade, date and time of measurement, clothes worn at the time of measurement, name and address of the school, body weight, height, waist circumference and hip circumference will be collected.
  - *Anthropometric measurements* shall be taken under standardized conditions and in the same order as presented in the examiner’s questionnaire.

The protocol set out in the Data Collection Procedure Manual developed specifically for the WHO European Childhood Obesity Surveillance Initiative (COSI) [2] shall be followed at all times.

In general, children should attend with normal, lightweight clothing. Before taking any of the measurements, they will be asked to remove their shoes and socks as well as any heavy clothing (coats, sweaters, jackets, etc.). They will also be asked to empty their pockets, to take off their belts or any other object and to remove any other accessories (headbands, pendants, etc.). Bodyweight will be measured in kg, up to the nearest 100 g. Height should be taken in cm, to the nearest mm. Moreover, a bioimpedanciometer (TANITA MC 780MA; Tanita corp.; Amsterdam; Netherland) will be used to evaluate fat mass, fat-free mass, lean mass, bone mass and muscle mass index.

1. **Family questionnaire:** This instrument collects information about the child’s lifestyle, eating habits, family health and sociodemographic data.
2. **School questionnaire:** This questionnaire includes questions related to physical activity, meals taken at school and access to food during school hours.

Physical condition. Assessing Levels of Physical Activity Battery (*ALPHA-fitness test battery)* [22,23]. The following indicators will be included: (a) upper-body strength (hand dynamometry); (b) lower-limbs strength (standing long jump); (c) cardiorespiratory component (Course-Navette test); and (d) speed-agility (4 × 10 m test). This battery will be applied to children and adolescents.

- *Handgrip strength*. Maximum handgrip strength will be measured by maximum hand dynamometry [24,25,26] using a digital dynamometer with an adjustable grip (TKK 5041 Grip D, Takei, Tokyo, 3.Japan). A table of estimated reference values will be used to adjust the grip [22].
- *Lower limbs strength.* Explosive lower-body strength will be assessed using the standing long jump with feet together (the longest distance possible). It will be measured in cm by means of a measuring tape (from the take-off line to the point where the back of the heel nearest to the take-off line lands on the ground) [27].
- *Cardiorespiratory fitness* will be assessed using the Course–Navette test (20-m shuttle run test). Participants will have to run between two lines separated by 20 m while keeping the rhythm emitted by audio signals. The initial speed is 8.5 km/h, which increased by 0.5 km/h every minute. Subjects should start when the audio signal or beep is heard. Participants were encouraged to continue running as long as possible during the test. The test finishes when the subject stops because of fatigue or fails to reach the end line concurrent with the audio signal or beep on two consecutive occasions [28,29]. The last half stage completed will be recorded for analysis.
- *Speed-agility* will be evaluated using the 4 × 10 m test [30], which consists of running a total distance of 40 m. Participants must run a distance back and forth between two lines 10 m apart, taking three sponges alternately as quickly as possible.

3. **Obesity and overweight.** They will be assessed through Body Mass Index (BMI) and fat percentage following to WHO methodology [2]. BMI will be calculated using the formula: weight (kg) divided by height squared (m^2^).
4. **Well-being.***The Perception Scale* of *Child Well-being Indicators (EPIBI)* will be used to assess well-being [31]. The original scale consists of 80-Likert scale items, including six different response categories (from 1 = not important to 6 = very important). These categories are grouped into five dimensions (material well-being, health and safety, educational well-being, relationships with the environment and subjective well-being). The student is asked to make a series of statements related to child well-being and to assess the degree of importance that every item supposes for the child’s well-being (Cronbach’s alpha = 0.95).

#### 2.4.2. Secondary Measures

##### Self-perceived Physical Fitness

The International Fitness Scale (IFIS) [32] will be used. This tool consists of 5-Likert scale questions about how participants perceive their overall physical fitness, cardio-respiratory fitness, muscle strength, speed-agility and flexibility. Response options are “very poor”, “poor”, “acceptable”, “good” and “very good” compared to their friends (Kappa = 0.45).

##### Physical Activity Adherence

Physical activity adherence will be assessed using the specific questionnaires from the International Physical Activity Questionnaire (IPAQ) for children and adolescents [33,34]:

- *Physical Activity Questionnaire for Adolescents (PAQ-A)* [35] consists of nine questions that assess different aspects of physical activity performed by adolescents in the previous 7 days, using a 5-point Likert scale for every question and reporting information about the intensity, frequency and duration of each activity performed. The overall result gives a score from 1 to 5, where a higher score indicates a higher level of physical activity. This questionnaire will be completed for adolescents aged 13 to 18 years old. Participants will complete the Spanish version (ICC = 0.71) [36].
- *The Physical Activity Questionnaire for Children (PAQ-C)* [37] is similar to the previous one but adapted to children aged between 8 and 14 years old (Cronbach’s alpha = 0.83).

##### Physical Literacy

The second version of the *Canadian Assessment of Physical Literacy Development (CAPL-2)* [38] will be applied. This tool is used to monitor the physical literacy of children. It combines assessments of daily activity behaviour, physical competence, motivation and confidence, and knowledge and understanding. CAPL-2 total score (100 points) will be calculated as the sum of daily behaviour (30 points), physical competence (30 points), motivation and confidence (30 points), and knowledge and understanding (10 points) domains. An algorithm proposed by the developer will be used to calculate the total CAPL score if any domain score is missing [38].

1. **Daily activity behaviour** will be objectively assessed from pedometer step counts during seven consecutive days. Also, children are asked to self-report the number of days in the last week that they were physically active for at least 60 min.
2. **Physical competence** will be assessed using the following three tests:
  - *Canadian Agility and Movement Skill Assessment (CAMSA)* [39] will consist of children to travel a total distance of 20 m while completing 7 movement skill tasks: (1) 2-footed jumping into and out of 3 hoops on the ground, (2) sliding from side to side over a 3-m distance, (3) catching a ball and then (4) throwing the ball at a wall target 5 m away, (5) skipping for 5 m, (6) 1-footed hopping in and out of 6 hoops on the ground and (7) kicking a soccer ball between 2 cones placed 5 m away. Groups of children will be instructed to complete the assessment as fast as possible while performing the skills to the best of their ability. Each child will be assessed bgy 2 timed and scored trials. The assessment will be administered and scored by two examiners. The first examiner will measure the completion time. The second examiner will evaluate the quality of each skill performed. The total score (maximum 10 points) will be calculated as the sum of the skill and time scores (completion time: ICC = 0.82; skill score: ICC = 0.74).
  - *Plank isometric hold* [40] is an isometric core strength exercise that involves maintaining a position similar to a push-up for the maximum possible time (inter-rater: ICC = 0.62; intra-rater: ICC = 0.83; test-retest: ICC = 0.63).
  - *Progressive Aerobic Cardiovascular Endurance Run (PACER)* [41] will be used to estimate the peak oxygen consumption in the youth. The participants will run 20 m back and forth between cones. The running pace will follow a sound signal. The initial speed will be beginning at 8.5 km/h and progressively will be increased by 0.5 km/h each minute. Participants must run as long as possible, and testing will stop when the participant reaches fatigue or cannot maintain the required pace for two consecutive times.
3. **Motivation and confidence** will be assessed by 12 self-report items. Four aspects of motivation and confidence are evaluated (predilection, adequacy, perceived competence satisfaction and intrinsic motivation). Predilection will assess the child’s preference for physically active pursuits. Adequacy will assess their expectations for success. Perceived competence satisfaction will assess whether children perceive they can complete optimally physical activities. Intrinsic motivation will assess if children pursue an activity for its own sake.
4. **Knowledge and understanding** will be measured using the *Physical Literacy Knowledge Questionnaire (PLKQ)* [42]. Questions will evaluate the children’s understanding of physical activity and sedentary behaviour recommendations, the awareness of fitness and movement skill parameters and the methods for their improvement, the perceptions of health and the use of safety equipment during activity (2-day interval: *r* = 0.62; 7-day intervals: *r* = 0.69).

##### Health-Related Quality of Life (HRQoL)

Health-related Quality of Life will be evaluated using the following questionnaires:

- *Kidscreen-52* [43]. These instruments assess children’s and adolescents’ subjective health and well-being. It is a self-report measure applicable for healthy and chronically ill children and adolescents aged from 8 to 18 years. The KIDSCREEN-52 measures 10-HRQoL dimensions: physical (5 items), psychological wellbeing (6 items), moods and emotions (7 items), self-perception (5 items), autonomy (5 items), parent relations and home life (6 items), social support and peers (6 items), school environment (6 items), social acceptance (bullying) (3 items) and financial resources (3 items). Its reduced version KIDSCREEN-10 will be also administered [44,45] (Cronbach’s Alpha = 0.82).
- *Child health utility 9D (CHU9D)* [46] is a self-report questionnaire (completed by the child) and a proxy-report questionnaire (completed by the caregiver). This questionnaire consists of 9 items that include 5 possible responses (scored 1–5) to assess the child or adolescent’s current status on the following domains: worry, sadness, pain, tiredness, annoyance, school, sleep, daily routine and activities.
- *Euroqol-5 Dimensions-Youth (EQ-5D-Y)* [47] consists of the EQ-5D-Y descriptive system and the EQ visual analogue scale (EQ-VAS). The descriptive system comprises the same estimated dimensions as Euroqol-5 Dimensions-3L (EQ-5D-3L), using a child-friendly wording. Each dimension has 3 levels: “no problems”, “some problems” and “a lot of problems”. Participant must indicate his/her health state by ticking in the box against the most appropriate statement in each of the 5 dimensions. The EQ-VAS is a self-rated visual analogue scale which records the respondent’s health, where the endpoints are labelled “the best health you can imagine” and “the worst health you can imagine”. Participants’ parents will complete a similar questionnaire named EQ-5D-Y Proxy (ICC > 0.7) [48].
- *16D* [49] is based on the 15D measure [50,51]. Some of the questions of the 15D measure were deleted (e.g., sexual life) and others were added (e.g., physical appearance and friends) or reformulated (e.g., usual activities) to be age-appropriate. This instrument is designed for participants aged 12–15 years. This tool will be translated to Spanish language following the author guidelines (already present in a signed agreement) prior to being administered to participants. *17D* [52] is based on the 16D [49] and 15D measures [50,51]. One of the questions measured in 16D was eliminated (distress) and others were added (e.g., ability to concentrate, learning and memory capacity and anxiety) or reformulated (e.g., vision and depression of vitality) to be appropriate for ages 7–11. This tool will be translated into Spanish language following the author guidelines (already present in a signed agreement) prior to being administered to participants.
- *AQoL-6D for adolescent* [53] is based on AQoL-6D [54,55]. This instrument includes 6 separately scored dimensions (independent living, relationships, mental health, coping, pain and senses), with variable item numbers and response levels.

##### Happiness and Satisfaction with Life

Happiness and satisfaction with life will be measured using the following three different scales:

- *Spanish Subjective Happiness Scale (SSHS)* [56]. This scale consists of a 4-item Likert scale that measures global subjective happiness by means of statements where participants self-rate themselves or compare to others. The scale has an adequate unitary structure and temporal stability confirmed in 14 samples (Cronbach’s alpha = 0.81).
- *Satisfaction with Life Scale (SWLS)* [57] is a multi-item scale as a measure of subjective well-being. This scale comprises 5 self-referencing statements on perceived global life satisfaction on a Likert scale ranging from 1 (strong disagreement) to 6 (strong agreement). Participants will complete the Spanish version of the SWLS [58].
- *Positive Affect Negative Affect Schedule (PANAS)* [59]. This questionnaire was constructed by Sandin (1997) [60] from the adult version by Watson et al. (1988) [61]. It is a 20-item self-report questionnaire. Ten items assess positive affect, and ten others assess negative affect. The questionnaire is completed by the child/adolescent considering the way he/she usually feels and/or behaves, following a scale of three response alternatives, described as “never”, “sometimes” and “many times”.

##### Connection with Nature

Connection with nature will be assessed using the following scales:

- *Connectedness to Nature Scale (CNS)* [62]. This is a self-administered questionnaire designed to assess an affective individual experience of connection with nature, which contains 14 items written in the form of a 5-point Likert-type scale. Participants will complete the Spanish version of the CNS (Cronbach’s alpha = 0.788) [63].
- *Nature Relatedness Scale (NR)* [64]. This is a self-report measure designed to assess the affective, cognitive and physical relationship individuals have with the natural world (Cronbach’s alpha = 0.87). Participants respond to statements using a 5-point Likert scale (1 = strongly disagree and 5 = strongly agree), and items are averaged with higher scores, indicating stronger connectedness. This tool will be translated into Spanish language following the WHO guidelines prior to be administered to participants [65].
- *Connection to Nature* [66]. The test consists of 18 multiple-choice questions which measure children’s attitudes toward the natural environment and which reflect the enjoyment of nature, empathy for its creatures, sense of oneness and sense of responsibility. The 18 items are written in the form of a 5-point Likert scale (1 = strongly disagree and 5 = strongly agree) (Cronbach’s α = 0.87). This instrument will be translated into Spanish language following the WHO guidelines prior to being administered to participants [65].

##### Self-Concept

This variable will be evaluated using the following instruments:

- *Physical Self-Description Questionnaire* [67]. This is a 70-item instrument designed to measure health, coordination, activity, body fat, sports competence, appearance, strength, flexibility, endurance and fitness, global physical self-concept and global self-confidence. Every scale is represented by six or eight simple declarative statement items, and participants answer using a 6-point true false scale. Participants will complete the Spanish version [68,69].
- *Figure Rating Scale* [70] consists of nine figures of increasing size with accompanying numerical ratings from 1 to 9. This body silhouettes method was designed and validated by Stunkard, Sørensen and Schulsinger [71]. It shows nine body silhouette figures of both men and women from very thin to very obese. The subjects should choose the figure which is closest to their own along with the silhouette they would like to have [72].

##### Barriers for the Physical Activity

- *Short Scale of Perception of Barriers for the Physical Activity in Adolescents* [73]. This is a 12-items self-reporting instrument that collects the self-perception about the different items included in the scale. Every item will be assessed using a 5-point Likert answer scale, where 1 means strongly disagree and 5 is strongly agree (Cronbach’s alpha = 0.80).
- *Self-Reported Barriers to Physical Activity (SBPA)* [74]. This is a 17-item questionnaire answered as a 10-points Likert scale, where values close to 0 mean that is very unlikely to prevent physical activity from being performed in the next weeks and values close to 10 mean very likely to prevent physical activity from being performed. This instrument consists of 4 factors: body image/physical and social anxiety; tiredness/laziness; life demands/lack of time; and environment/facilities (Cronbach’s alpha = 0.85).

##### Bullying

This variable will be measured by two different questionnaires:

- *Bull-S questionnaire* [75] assesses three general aspects of bullying: (1) sociometric position (items 1–4), which evaluates how each person is positioned by his or her status into the group and what the social and affective structure of the group is like; (2) bullying characteristics (items 5–10) that assess some physical and personal aspects of bullies or to victims; and (3) situational properties (items 11–15) that use a peer-report procedure to estimate the type, place and frequency of the aggressions as well as the degree of severity that pupils confer on this behaviour and how safe they feel at school. Participants will complete the Spanish version [76,77] (Cronbach’s alpha = 0.73).
- *School Violence Questionnaire-Revised (CUVE-R)* [78]. This is a revised and expanded version of the CUVE [79] (Cronbach’s alpha = 0.92). CUVE-R evaluates the students’ perception of the frequency of the emergence of different types of school violence involving students and teachers. It consists of a 5-point Likert scale with 31 items. Participants must indicate when the violent incident occurred (1 = not occurs never; 2 = occurs rarely; 3 = sometimes; 4 = many times; and 5 = always). This instrument will be translated into Spanish language following the WHO guidelines prior to being administered to participants [65].

### 2.5. Statistical Analysis

All the information collected will be tabulated in a database designed for this purpose using IBM SPSS Statistics 25 (participants data will be anonymous). Baseline characteristics of participants will be presented as mean (standard deviation) for continuous variables and proportions for categorical variables. Normality will be checked using the Kolmogorov–Smirnov test. The criteria established in the COSI initiative will be followed, and the WHO growth standards will be used to establish the different situations of “normoweight”, “overweight” and “obesity”. The weight situation of each child/adolescent will be established based on the following criteria based on the standard deviation (SD): 1) “severe thinness” BMI < −3 SD; 2) “thinness” BMI < −2 SD; 3) “overweight” BMI > +1 SD; and “obesity” BMI > +2 SD. To compare our results with other previous studies, the weight situation will be assessed considering the International Obesity Task Force (IOFT) criteria [80] and the growth curves of the Orbegozo Foundation. The waist to hip ratio (WHpR) and the waist to stature ratio (WSR) will be calculated to establish the degree of central adiposity. A WSR > 0.5 will be considered as high. A 95% confidence interval will be calculated using mean values and proportions. The Chi-square test will be used to check the association between qualitative variables, and the Student T-test or U Mann–Whitney test (if the distribution is not normal) shall be used to analyse between-group differences. If the analysis includes more than two groups, an ANOVA or Kruskal–Wallis test (if non-normal data distribution) will be applied. Significant differences are considered for *p* ≤ 0.05.

## 3. Discussion

To our knowledge, this would be the first study that would show the specificity, update and epidemiological data of overweight, obesity, motricity and well-being in children and adolescents aged from 6 to 17 years old in Extremadura.

As mentioned above, the Global Matrix [6], ALADINO [3] and PASOS [5] studies present data at a national level, without between-region differentiation. Moreover, the Global Matrix and ALADINO studies only cover a very small age range (6–9 years) [3,17].

Global Matrix highlights that the main limitation of its 2018 Report Card is the reliance on data obtained from subjective methods for assessing physical activity and the different types of questionnaires used. Thus, they indicate that it is quite difficult making a comparison across surveys and studies because data from physical fitness, community and environment, family and peers, and government indicators are not reported. Nevertheless, they justify this limitation by clarifying that Spain has 17 very different autonomous regions, and the periodic data on physical activity is limited in most of them. Considering this Spain’s Autonomous Communities diversity and HDI, we find that Extremadura presents a lower HDI compared to the average HDI of Spain, being only higher in comparison with Ceuta and Melilla [81]. For example, we found a 0.847 vs 0.885 HDI in 2015 [82]. Therefore, this project will try to complement and complete the data required by the Global Matrix in Extremadura as well as to relate them to other indicators, such as the overweight and obesity level or well-being in children and youth.

There are many previous studies that have shown the positive effect of different fitness interventions on obesity patients [83]. Many of them have related physical fitness and/or overweight to different aspects, such as self-concept [84], bullying [85], emotional well-being [86], barriers to physical activity [87] or HRQoL [88]. However, no studies have evaluated and interrelated all the aspects mentioned above and their influence on overweight, motricity and well-being in children and youth, using the results obtained to guide possible multidisciplinary interventions and to monitor them.

Thus, the obtained results will be important to determine whether children and youth in Extremadura are within the recommended values and, if not, to be able to undertake the pertinent interventions to reverse this situation. These outcomes would represent key elements contemplated into some models of “Health Promotion and Disease Prevention Toolkit”, such as the Health Belief Model, and could be useful to design or adjust health promotion or disease prevention programs, alone or in combination with other theories or models [21]. Likewise, this observatory will analyse the impact and process of the policies adopted so far, according to the Policy, Regulatory, and Organizational Constructs in Educational and Environmental Development model (PROCEED) standards [20] by looking at which interventions have obtained the best results as well as the success of subsequent interventions. Additionally, from the evaluation of Global Matrix indicators, we will be able to know the level of Extremadura and to compare this data with other countries that present better results in order to guide the policies to be adopted.

From a business point of view, the results of this study will suppose an opportunity, since parents may be interested in knowing the situation of their child regarding his/her health status and be willing to hire a service that includes the evaluation and monitoring of these parameters, along with an intervention program. In addition, collaborations could be established with different companies interested in this project which obtain reference data that can be then used to classify and compare future users of the overweight and obesity monitoring service. Thus, it will be possible to design a business service to satisfy the needs found through the observatory and to design novel strategies to prevent and reduce the impact of overweight and obesity as well as their derived consequences.

## 4. Conclusions

This project will aim to monitor, evaluate and interrelate physical, social, psychological and health variables using the obtained results to manage and propose possible multidisciplinary interventions and strategies in order to prevent and reduce the overweight and obesity problem in children and youth. Also, the results of this study will be important to determine whether children and youth of Extremadura comply with recommended values and to be able to undertake the pertinent interventions to attend this situation. Moreover, orientations will be given to transfer the obtained results to the public sector to evaluate or change the adopted policies and to the business sector.

## Figures and Tables

**Table 1:** Assessments schedule.

Assessments		Year 1	Year 2	Year 3	Year n
Main Measures	COSI initiative measures	X	X	X	X
	ALADINO study measures	X	X	X	X
	ALPHA-fitness test battery	X	X	X	X
	Obesity and overweight	X	X	X	X
	Well-being	X	X	X	X
Secondary Measures	Self-perceived physical fitness.	X	X	X	X
	Physical activity levels	X	X	X	X
	Physical literacy	X	X	X	X
	Health-Related Quality of Life (HRQoL)	X	X	X	X
	Happiness and satisfaction with life	X	X	X	X
	Connection with nature	X	X	X	X
	Self-concept	X	X	X	X
	Barriers for the Physical Activity	X	X	X	X
	Bullying	X	X	X	X

COSI: Childhood Obesity Surveillance Initiative; ALADINO: Growth Monitoring, Nutrition, Physical Activity, Child Development and Obesity Study in Spain; ALPHA-Fitness: Assessing Levels of Physical Activity Battery.
